# Visceral leishmaniasis in eastern Africa – current status

**DOI:** 10.1016/j.trstmh.2007.06.001

**Published:** 2007-12

**Authors:** Richard Reithinger, Simon Brooker, Jan H. Kolaczinski

**Affiliations:** a Westat, Rockville, USA; b London School of Hygiene and Tropical Medicine, London, UK; c George Washington University, Washington D.C., USA; d Malaria Consortium Africa, Sturrock Road, Kampala, Uganda

**Keywords:** Leishmaniasis, Kala-azar, Sandflies, Prevention, Control, Africa

## Abstract

Visceral leishmaniasis (VL) is among the most neglected of the tropical diseases, afflicting the poorest of the poor. In eastern Africa, VL causes at least 4000 deaths annually, a loss of approximately 385 000 disability-adjusted life years. Due to the chronicity of underlying causes, it is likely that the caseload will increase in the foreseeable future. While efforts should be pursued to develop novel case management and prevention tools, several effective interventions already exist but are rarely deployed. Funds are needed now to procure commodities and strengthen health systems, so that effective VL control can be delivered to populations at risk.

Visceral leishmaniasis (VL), or ‘kala-azar’, is a systemic disease characterised by a range of symptoms including fever, (hepato)splenomegaly, lymphadenopathy, weight loss, weakness and, if left untreated, death (Murray et al., 2005). VL is usually caused by *Leishmania donovani* or *L*. *infantum*, protozoan parasites that are transmitted by phlebotomine sandflies when feeding on the blood of human and animal hosts. Eastern Africa is one of the world's main VL-endemic areas, and the disease occurs in numerous foci in Eritrea, Ethiopia, Kenya, Somalia, Sudan and Uganda.

The number of VL cases in the region has increased dramatically in the past decade. A major contributing factor has been the ongoing armed conflicts, which have caused widespread destruction of local housing and health infrastructures, increasing people's exposure to sandfly bites and making it hard to manage cases and deliver vector control activities. The conflicts have, in turn, induced massive movements of susceptible or infected populations into VL-endemic or non-endemic areas, respectively, triggering major epidemics. Added to this flux, sustained droughts have lead to widespread malnutrition and famine, two of the main risk factors for VL. Conflict and drought probably caused the largest VL epidemic ever recorded, during the 1980s in Sudan, where VL killed an estimated 100 000 people (Seaman et al., 1996), a figure which may even be considerably higher because of substantial under-reporting of VL (Collin et al., 2006). A final factor contributing to the rise in VL cases in some populations is the increasing prevalence of HIV (Anema and Ritmeijer, 2005), another common risk factor for VL (Murray et al., 2005). Because mass migration of VL-affected populations due to either armed conflict (e.g. Somalia) or peace (e.g. Sudan) will continue over the coming years, it is likely that the caseload will increase even further. Despite the significant and increasing VL burden in eastern Africa, efforts to control the disease are either non-existent or remain sub-optimal.

Few VL-endemic countries in eastern Africa have functional leishmaniasis control programmes, and these have little or no resources to train health staff on VL case management and methods for prevention and control, let alone to procure commodities to implement activities. Many affected people thus have no access to VL diagnosis, treatment or prevention, and they die, undetected by the health information system, in their community (Collin et al., 2006). Those cases that receive VL treatment often do so only because of temporary health service delivery provided by third party organisations (e.g. Médécins Sans Frontières) or international research projects (e.g. through the Leishmaniasis East African Platform of the Drugs for Neglected Diseases Initiative). Moreover, owing to difficulties in accessing endemic foci (e.g. because of security concerns or lack of infrastructure) up-to-date and scientifically credible data on the epidemiology, disease burden and possible risk factors of VL are scarce. The lack of such information adds to the difficulty of identifying and targeting populations at risk, and selecting appropriate tools for prevention and control.

However, several effective tools to diagnose, treat or prevent VL have been developed and tested (Murray et al., 2005), including: highly sensitive and specific rapid immunochromatographic dipsticks; effective and safe generic antimonial drugs; and long-lasting insecticidal nets (LLINs) for personal protection. Other tools have yet to be fully developed, evaluated or operationalised, particularly for eastern Africa; these include the use of geographic information systems and remote sensing data to delineate VL risk areas, and the use of drugs other than antimony to reduce treatment duration, cost and toxicity and to delay antimony drug resistance.

Rather than wait for the results from ongoing studies on novel tools, the time has come to deploy the existing ones. We advocate a strategy that combines commodity support (e.g. for dipsticks, drugs and LLINs), health system strengthening, capacity building and operational research. Wherever feasible, interventions should be integrated into multifunctional health service delivery in order to maximise effectiveness and resources. If substantial funds were made available to provide this support, the reduction of the VL burden would be highly cost-effective (Laxminarayan et al., 2006). Improved estimates of the number and location of people at risk are essential to determine and target intervention resources required to control or even eliminate VL, and to lobby for the increased financial and political support to do so. Limited support is currently being provided by WHO and, primarily, non-governmental organisations to help establish and support control programmes in Ethiopia, Kenya and Sudan. There remains a need to expand these efforts, ensuring a coordinated regional approach.

Past experience has shown that VL has a devastating morbidity and mortality impact on affected communities. Several tools to effectively prevent infection and manage VL exist; however, there has been a chronic lack of funds to effectively implement them operationally. Making these tools available at a greater scale, both locally and regionally, will lead to significant inroads into the disease's incidence and distribution.

## Funding

The authors’ work on VL in Uganda is being supported by the Sir Halley Stewart Trust. SB is supported by a Wellcome Trust Career Development Fellowship.

## Conflicts of interest

None declared.

## Ethical approval

Not required.
